# Age-associated decrease in de novo donor-specific antibodies in renal transplant recipients reflects changing humoral immunity

**DOI:** 10.1186/s12979-019-0149-8

**Published:** 2019-05-09

**Authors:** Seraina von Moos, Gesa Schalk, Thomas F. Mueller, Guido Laube

**Affiliations:** 1 Department of Nephrology, University of Zurich and University Hospital Zurich, Rämistrasse 100, 8091 Zürich, Switzerland; 2 Department of Nephrology, University of Zurich and University Children’s Hospital, Steinwiesstrasse 75, 8032 Zurich, Switzerland

**Keywords:** De novo donor specific antibodies, Aging, Older kidney transplant recipients, Pediatric kidney transplant recipients, Immunosenescence

## Abstract

**Background:**

Older age at organ transplantation is associated with increased risk of infection and malignancy but reduced risk of cellular rejection. De novo donor-specific anti-HLA antibodies (*dn*DSA), are key biomarkers associated with reduced long-term allograft survival, yet there is a lack of data focusing on age-associated changes.

**Methods:**

Development of *dn*DSA was restrospectively analyzed in all subjects who received a kidney transplant at the University Hospital Zurich between 01/2006 and 02/2015. Follow up continued until 03/2016. The incidence of *dn*DSA in different age categories was compared with special focus on the extremes of age: children < 10 years (*n* = 19) and adults ≥60 years of age (*n* = 110).

**Results:**

Incidence of *dn*DSA gradually decreased with age, with older recipients having a significantly lower risk (HR 0.21, *p* = 0.0224) compared to pediatric recipients. Cumulative incidence of *dn*DSA at 2, 5 and 10 years was 6.2, 9.1 and 36% in the older recipients versus 5.3, 29.5 and 47.1% in pediatric recipients. Median time to development of *dn*DSA was similar (older 720 days, min 356, max 3646 days; children 1086 days, min 42, max 2474 days). Annual incidence was highest within the first two years after transplantation in the older recipients and peaked in years two to four in pediatric recipients. *Dn*DSA were predominantly class II. More *dn*DSA were observed with cyclosporine as compared to tacrolimus.

**Conclusion:**

Older kidney transplant recipients have a lower risk of developing *dn*DSA than pediatric recipients, pointing towards reduced humoral immune reactivity with increasing age. This observation raises the question of adjustment in immunosuppression.

**Electronic supplementary material:**

The online version of this article (10.1186/s12979-019-0149-8) contains supplementary material, which is available to authorized users.

## Introduction

In recent years, interest in the changing immune reactivity over the life course has increased, as the number of older transplant recipients is steadily rising [1, 2]. Consideration of changing immune reactivity with increasing age, generally known as inflamm-aging and immunosenescence [3–6], is essential in the unique context of organ transplantation. While inflamm-aging collectively refers to the increased level of stimulation of the innate immune system, the concept of immunosenescence refers to alterations in the adaptive immune system with decreased numbers of naïve T cells. The reduced ability to mount immune responses against novel antigens with increasing age is associated with increased risks of infection and malignancy and reduced vaccination responsiveness [3]. Particularly in the setting of organ transplantation, where the risk of rejection needs to be balanced with the risk of infections and malignancies [7], this changing immune reactivity with age should be considered in order to optimize immunosuppressive drug dosing [8]. In the field of organ transplantation, most studies comparing old with young transplant recipients have focused on T-cell responses [9] and have indeed described reduced frequency of acute T-cell mediated rejections in older kidney transplant recipients as compared to pediatric recipients [10, 11]. Few studies have investigated antibody responses, i.e. development of *dn*DSA against specific HLA-antigens despite increasing recognition of the key role for antibody-mediated rejection on long-term graft survival [12, 13]. In addition, existing studies almost exclusively associate aging with old age and studies on immune responses in pediatric recipients are scarce. This study focuses on the changing immune reactivity of the humoral immune responses in kidney transplant recipients, comparing the incidence of *dn*DSA in different age groups with a special focus on the those under 10 years and over 60 years of age.

## Methods

### Patient population: inclusion and exclusion criteria

In this longitudinal retrospective analysis all patients receiving a first single kidney transplant at the University Hospital of Zurich between January 2006 and February 2015 were eligible for inclusion. Follow up continued until March 2016. In depth analysis of changing immune-reactivity was performed for all children < 10 years of age and for adults ≥60 years of age.

In order to minimize bias from medication non-adherence known to be up to 43% in adolescent transplant recipients as compared to 22% in children and adults, we defined the pediatric reference cohort as children younger than 10 years of age [14–16]. As biological aging represents a spectrum, no clear clinical cut off for age has been defined for older transplant recipients. Changes associated with immunosenescence are described to start by 50 years of age [9, 17, 18] and by general convention organ donors older than 60 years of age are considered extended criteria donors, due to differences in immunogenicity of older donor organs [10, 19]. We therefore selected 60 years of age to define the older transplant recipient cohort [9]. Patients were excluded if the following were present: pre-existing DSA, missing donor typing information on DQ locus and possible DSA in the Luminex single bead assay (SAB) according to HLA associations, DSA MFI levels below 1000 in the Luminex SAB and patients with incomplete follow up data. The study was approved by the local ethics committee of Zurich (protocol number: KEK-ZH-Nr. 2017–00500).

### Follow up of transplant patients, immunosuppression regimens and Luminex assays

During the first year after transplantation, patients were followed closely in the transplant clinic. Thereafter, patients were seen at least yearly. Induction therapy with basiliximab or thymoglobulin was used in patients at high risk for rejection. Maintenance immunosuppression consisted mostly of a calcineurin inhibitor (cyclosporine A or tacrolimus) and an anti-proliferative drug (generally mycophenolic acid). Steroids were withdrawn 6 months after transplantation in the majority of patients. Target trough levels at 6, 12 and 24 months were 100-160 ng/ml, 80-120 ng/ml, 50-80 ng/ml and 7-10 ng/ml, 6-8 ng/ml, 4-6 ng/ml, for cyclosporine and tacrolimus respectively. When the post-transplant course was uncomplicated, HLA-specific antibody testing was done yearly. With graft dysfunction additional HLA-specific antibody testing was performed. Testing was done using LABScreen Mix Class I and II antibody screening kit (OneLambda, Canoga Park, CA). If the Luminex mix screening assay was positive, a specific Class I and/or Class II LAB Screen single-antigen assay was added (OneLambda, Canoga Park, CA). The data was analyzed using the kit specific HLA Fusion software (One Lambda). Antibodies to all loci (HLA-A, B- C, DR, DP and DQ) were included. Donor and recipients were typed for HLA-A, B, DR and from 2012 onwards for DQ). Typing was done using PCR-SSP based molecular typing method (Protrans, Mannheim, Germany) [20]. Calculated mean fluorescence intensity (MFI) values were normalized against the internal negative control and the negative serum control. If an allele specific antibody against the donor was discovered in the single antigen Luminex screening assay, it was declared as DSA. A cutoff of 1000 MFI was chosen according to the Swiss Organ Procurement System (SOAS). The DSA with the highest MFI was defined as the peak DSA.

### Statistical analysis

Occurrence of *dn*DSA after transplantation in the two age groups was analyzed using Kaplan Meier curves. The log rank test was used for curve comparison. Peak MFI of *dn*DSA was compared between the two groups with Mann Whitney U Test. The Chi square test was used to compare immunosuppressive medication in patients with and without development of *dn*DSA. Graph Pad Prism 5 Software were used for statistical analyses.

## Results

### Patient cohort

**Fig. 1:**
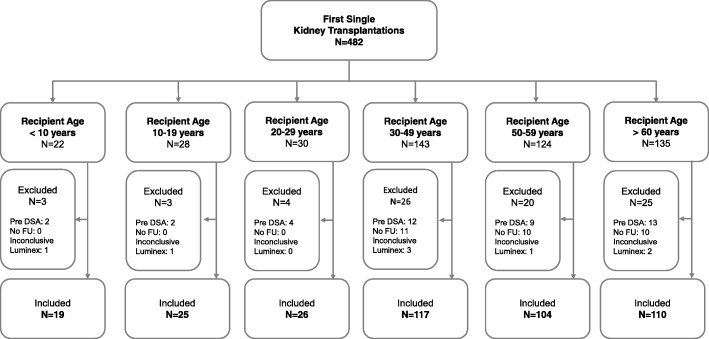
Study Flow Chart

Of 482 patients who received a single first kidney transplant between January 2006 and February 2015, 401 patients met inclusion criteria. A detailed patient flow chart is shown in Fig. 1. Patient distribution into different age groups and baseline characteristics are reported in Table 1. Median follow-up time did not significantly differ between the different age groups (*p* = 0.54; Table 1). The average number of mismatches per locus for the extremes of age groups are indicated in Additional file 1: Table S1.

**Table 1:** Study population

	Children < 10y	Adolescent 10-19y	20-29y	30-49y	50-59y	Old ≥ 60y
Total number	19	25	26	117	104	110
Mean age at transplantation	6 ± 3	15 ± 3	25 ± 3	43 ± 5	56 ± 3	65 ± 4
Male gender, n (%)	10 (53%)	17 (68%)	16 (62%)	81 (69%)	66 (63%)	83 (75%)
Deceased transplant n (%)	11 (58%)	16 (64%)	3 (12%)	59 (50%)	60 (58%)	76 (69%)
Median follow up time, days (min; max)	1971 (356; 3420)	1405 (289; 3427)	1788 (343; 3444)	1790 (157; 3645)	1841 (154; 3624)	1602 (141; 3612)

HLA mismatches were calculated for loci HLA A, B (class I) and DR, DQ (class II)

### Gradual decrease of de novo DSA with increasing recipient age

**Fig. 2:**
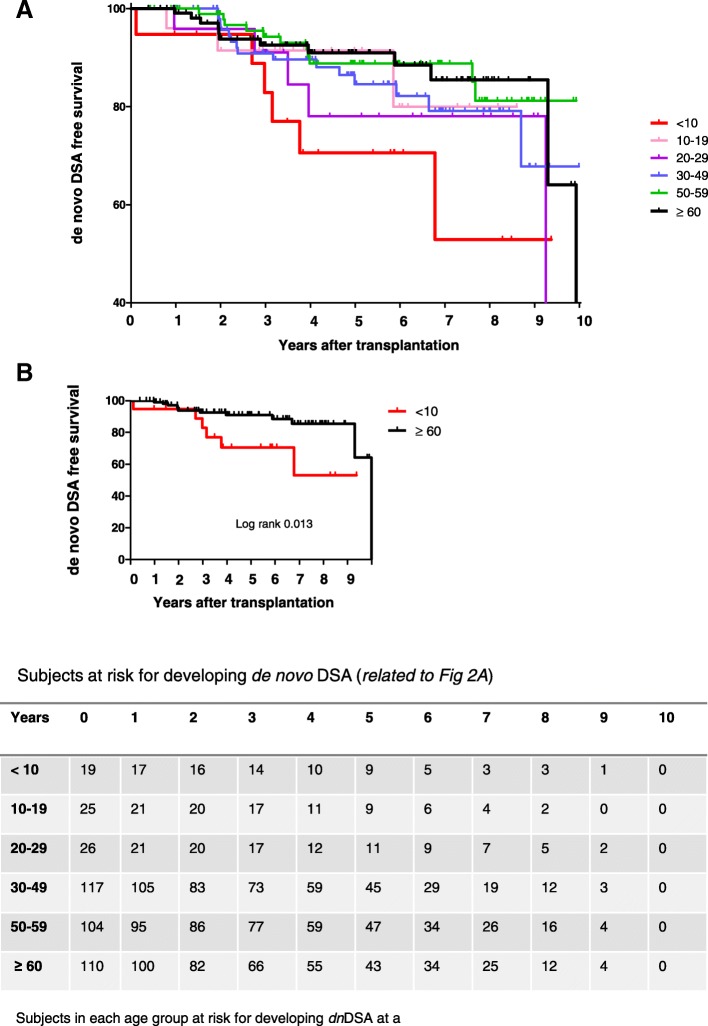
Cumulative incidence of *dn*DSA after kidney transplantation as determined by Kaplan Meier survival curves. **a** Comparison between all age groups, **b**) Comparison between the older recipient cohort and the pediatric recipient cohort using Log rank test. Subjects at risk for developing *dn*DSA in each time period are shown

**Fig. 3:**
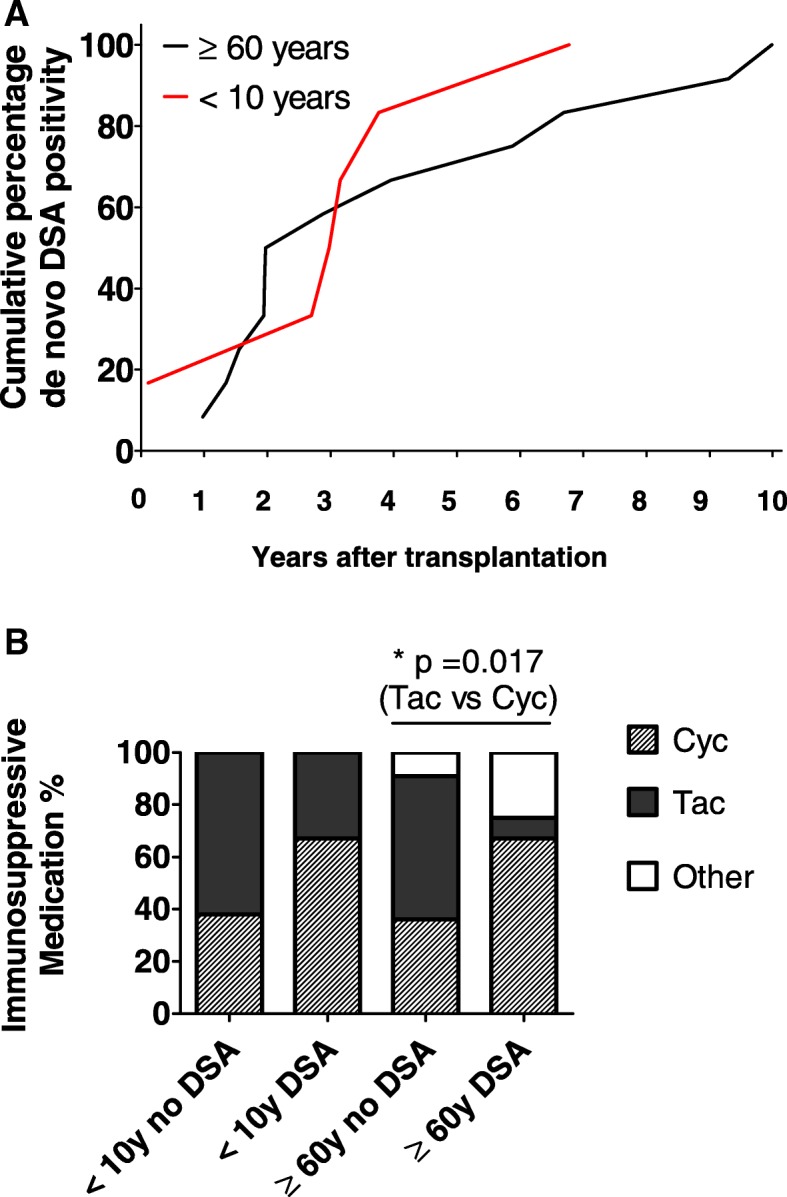
**a** Cumulative percentage of de novo DSA positivity in the cohort of recipients developing a donor specific humoral immune response: *n* = 12 in the older cohort and *n* = 6 in the pediatric cohort. **b** Comparison of immunosuppressive medication (Cyclosporine = Cyc, Tacrolimus = Tac) at time point of detection of de novo DSA between recipients with and without de novo donor specific humoral immune response in both cohorts, i.e. older and pediatric patients. Comparison using Chi-square test

A significantly lower risk for development of *dn*DSA was observed in transplant recipients in the age groups 50–59 years (HR 0.18; *p* = 0.014) and ≥ 60 years of age (HR 0.21, *p* = 0.22) as compared to children (< 10 years of age). Risk of developing *Dn*DSA was not significantly higher among adolescents compared with younger patients (HR 0.42, *p* = 0.20). Overall, a tendency towards decreasing risk for development of *dn*DSA with increasing age was observed, Fig. 2a. Comparison between the two extremes of age is highlighted in Fig. 2b. Cumulative prevalence of *dn*DSA for the different age groups is shown in Table 2. Further indepth analysis focused on the age extremes comparing older transplant recipients ≥60 years (i.e. older recipient cohort) and children < 10 years (i.e. pediatric recipient cohort). Cumulative incidence of *dn*DSA at 2, 5 and 10 years for the older cohort was 6.2, 9.1 and 36% as compared to 5.3, 29.5 and 47.1% in the pediatric cohort. Annual incidence of *dn*DSA was highest in the first two years after transplantation for the older recipients, whereas annual incidence peaked between two and four years after transplantation in the pediatric recipients (Fig. 3a). Yet, median time to development of DSA was similar in the older recipient cohort (720 days; min 356, max 3646 days) and in the pediatric recipient cohort (1086 days; min 42, max 2474 days). Mean age at occurrence of *dn*DSA was 71 years in the older recipient cohort and 8 years in the pediatric recipient cohort. Peak DSA was mostly a class II HLA antibody in both recipient cohorts (5 out of 6 in pediatric recipients; 9 out of 12 in older recipients), Additional file 1: Table S2. MFI of the peak *dn*DSA was not statistically different between the pediatric recipients (mean peak MFI 6408) and the older recipients (mean peak MFI 7023, Additional file 1: Figure S1). Of note, similar percentages of patients displayed *dn*DSA with MFI > 5000 and with MFI > 10′000 respectively (pediatric recipients: 3/6 with *dn*DSA MFI > 5000 and 2 out of these with MFI > 10′000, older recipients: 6/12 with *dn*DSA MFI > 5000 and 4 out of these with MFI > 10′000).

**Table 2:** Risk of development of *dn*DSA in different age groups

	Children < 10y	Adolescent 10-19y	20-29y	30-49y	50-59y	Old ≥ 60y
Cumulative prevalence dnDSA	32% (6/19)	12% (3/25)	19% (5/26)	13% (15/117)	11% (11/104)	11% (12/110)
Hazard ratio, p	–	HR 0.42 *p* = 0.205	HR 0.52 *p* = 0.312	HR 0.35 *p* = 0.088	HR 0.18 p = 0.014	HR 0.21 p = 0.022

### Immunosuppression

*Dn*DSA developed significantly more often among older recipients receiving cyclosporine- compared with tacrolimus-based regimens (Fig. 3b and Additional file 1: Table S3). A similar trend was observed in pediatric recipients, which did not reach statistical significance.

## Discussion

Due to demographic changes, increasing numbers of patients aged over 60 years are being transplanted [1, 2]. Hence, investigation of changing immune reactivity over the life course is increasing. In general, older recipient age is an independent risk factor for post-transplant mortality in all solid organ transplantations [9, 21], with the highest risk being infection-associated death followed by cardiovascular death and malignancies [22]. In contrast, the risk of acute cellular rejection is lower in older transplants recipient compared with pediatric recipients [23, 24]. These observations reflect changes in the adaptive immune system summarized by the terms ‘immunosenescence’ and ‘inflamm-aging’. The reduced risk of acute cellular rejections is consistent with thymic involution and the limited T-cell receptor repertoire observed with aging [6, 9, 21]. Additionally, humoral immune responses in older patients have also been shown to be altered, with increased memory responses and a skewed B cell repertoire [11, 25, 26] which is more specialized but less plastic to mount humoral immune responses. Together with the reduced frequency of naïve T cells these changes are associated with increased risk of infections [11, 26]. By contrast, the heightened subclinical inflammation associated with inflamm-aging and increased reactivity of the innate immune system potentially contributes to increased cardiovascular risk among older transplant recipients. Together, these aspects highlight the complex nature of immunological changes associated with aging.

Given the major role of antibody-mediated responses in determining long-term graft survival [12, 13], we analyzed changes in humoral immune reactivity with increasing age by investigating the incidence of *dn*DSA in older kidney transplant recipients compared with pediatric kidney transplant recipients. Our results indicate that the risk of developing *dn*DSA is 4.8 to 5.6 times lower in older recipients as compared to pediatric recipients (older aged ≥60 years HR 0.21; *p* = 0.0224). Interestingly, we observed that the changing reactivity of the immune system may begin around age 50 years (HR 0.18, *p* = 0.014). Of note, the annual incidence of *dn*DSA was highest during the first two years post-transplantation in the older recipients, while the annual incidence of *dn*DSA peaked between years two and four after transplantation in pediatric recipients These early post-transplant years are a time when immunosuppressive drug doses are generally reduced. Consistent with the literature, the majority of *dn*DSA were directed against HLA class II, more specifically against HLA DQ locus [27, 28] and *dn*DSA occurred significantly more frequently with cyclosporine- rather than tacrolimus-based immunosuppression [22].

To our knowledge, this is the first study directly comparing the incidence of *dn*DSA in older vs. pediatric kidney transplant recipients. Few small studies have investigated the development of *dn*DSA in older patients from the European senior program, reporting variable cumulative prevalences for *dn*DSA between 20 and 30% at 5 to 10 years post transplantation [22, 29], numbers that are higher than those observed in our cohort. Yet, development of *dn*DSA after kidney transplantation is highly variable with reports ranging from cumulative prevalences from 13 to 30% in non-sensitized adult kidney transplant recipients [30, 31]. This variability could reflect differences in antibody MFI cut offs, frequency of testing and baseline immunosuppression. With respect to the young cohort, our data are consistent with results from other centers reporting a cumulative prevalence between 20 and 36% at 5 to 10 years after transplantation [27, 32, 33].

A major strength of our study is the direct comparison between the different age groups of transplant recipients treated and followed up at the same hospital and tested for development of *dn*DSA in the same laboratory. To our knowledge this is the first study to directly compare development of *dn*DSA across patient age groups simultaneously and is therefore novel. We specifically set the age limit for definition of the pediatric recipient cohort at age younger than 10 to minimize the confounder of non-adherence, which is a relevant problem in adolescent recipients [34–36]. All but one pediatric recipient developed *dn*DSA at age ≤ 10 years of age (Additional file 1: Table S4). Hence, the differences in incidence and prevalence of *dn*DSA are most likely due to age-associated changes in immune reactivity and do not reflect differences in medication adherence. Assessment of medication adherence was however not possible in this retrospective study. The study does have several limitations. Subject numbers included in the extremes of age groups were different. Given the retrospective nature of the study, data regarding C1q binding of the *dn*DSA was not available. We did not investigate the effect of donor-recipient age ratio, which is another limitation given the known effect of organ age on immune reactivity [10, 19]. Additionally, at our center, we do not perform protocol biopsies, making a statement with respect to histological changes related to development of *dn*DSA impossible. Similarly, due to small patient numbers, data on graft survival would not be meaningful and in older adults is also highly impacted by comorbidities and not only rejection episodes [37]. Also, chronological age might not be congruent with biological age, considering the multiple modifying extrinsic factors such as nutrition, exercise and previous exposure to microorganisms [5] which adds to the complexity of changing pharmacodynamics and pharmacokinetics of immunosuppressive drugs with age [38]. Pharmacokinetic studies were not possible in this retrospective analysis.

In conclusion, this study adds to current knowledge having investigated humoral immune responses after kidney transplantation over the whole age spectrum. A gradual decrease in incidence of *dn*DSA, with age was observed, underscored by a significantly lower risk of *dn*DSA in the older recipient cohort as compared to the pediatric recipient cohort. Our observations may have practical implications with respect to immunosuppressive medication dosing and suggest that medication levels should be tailored to the individual needs reflecting the adaptive changes of immune reactivity associated with aging. Further prospective studies, including larger patient numbers and recipients of different solid organ transplants, with monitoring of medication adherence, including pharmacodynamic and pharmacokinetic studies as well as biomarkers associated with biological aging [3, 4, 39] are needed to confirm our data and to introduce a practical guidance on immunosuppressive medication dosing in accordance to transplant recipient age.

## Additional file

Additional file 1: **Figure S1.** Comparison of MFI Peak de novo DSA. **Table S1.** Average Mismatches. **Table S2.** Peak *dn*DSA Specifity for the individual patients. **Table S3.** Immunosuppression. **Table S4.** Individual characteristics of the pediatric recipients developing de novo DSA. (DOCX 35 kb)

## Abbreviations

*dn*DSA: de novo donor specific antibodies
HLA: Human leukocyte antigen
Luminex SAB: Luminex single antigen bead assay
MFI: Mean fluorescence intensity
