# Supplementary material for: Age-associated decrease in de novo donor-specific antibodies in renal transplant recipients reflects changing humoral immunity
Source: Immun Ageing. 2019 May 9;16:9. doi: 10.1186/s12979-019-0149-8 (PMC6509825; doi:10.1186/s12979-019-0149-8)
Supplement: Supplementary file 1 — Figure S1. Comparison of MFI Peak de novo DSA. Table S1. Average Mismatches. Table S2. Peak dnDSA Specifity for the individual patients. Table S3. Immunosuppression. Table S4. Individual characteristics of the pediatric recipients developing de novo DSA. (DOCX 35 kb) [file 12979_2019_149_MOESM1_ESM.docx]

Supplementary Figure 1: Comparison of MFI Peak *de novo* DSA

Supplementary Figure 1

Comparison of MFI of the peak *de novo* DSA in the elderly and the pediatric cohort using Mann Whitney U test.

Suppl Table 1: Average Mismatches

| Mismatch loci | CHILDREN (< 10 years) | OLD (≥60 years) |
| --- | --- | --- |
| HLA-A | 1.73 | 1.48 |
| HLA-B | 1.91 | 1.52 |
| HLA-DR | 1.31 | 1.12 |
| HLA-DQ | 1.45 | 1.09 |

Suppl Table 2: Peak *dn*DSA Specifity for the individual patients

| CHILDREN (< 10 years) | OLD (≥60 years) |
| --- | --- |
| Patient 1: DQ2 | Patient 1: DQ7 |
| Patient 2: DQ6 | Patient 2: DQ7 |
| Patient 3: A2 | Patient 3: DR53 |
| Patient 4: DQ2 | Patient 4: DQ7 |
| Patient 5: DR4 | Patient 5: A1 |
| Patient 6: DQ2 | Patient 6: B8 |
|  | Patient 7: DR4 |
|  | Patient 8: A26 |
|  | Patient 9: DR53 |
|  | Patient 10: DQ2 |
|  | Patient 11: DQ4 |
|  | Patient 12: DQ6 |

Suppl. Table 3. Immunosuppression

|  | CHILDREN (n=19) | | | OLD (n=110) | | |
| --- | --- | --- | --- | --- | --- | --- |
|  | No DSA  (n=13) | *De novo* DSA  (n=6) | | (n=98) | *De novo* DSA  (n=12) | |
|  | At last FU | Before DSA | At last FU | At last FU | Before DSA | At last FU |
| Tac/MMF | 8 | 2 | 4 | 35 | 1 | 2 |
| Tac/MMF/P |  |  |  | 14 |  | 2 |
| Tac/Imurek |  |  |  | 1 |  |  |
| Tac/Imurek/P |  |  |  | 4 |  |  |
| Tac + x, n (%) | 8 (61.5) | 2 (33.3) | 4 (66.7) | 54 (55.1) | 1 (8) | 4 (33.3) |
| Cyc/MMF | 5 | 4 | 2 | 23 | 8 | 5 |
| Cyc/MMF/P |  |  |  | 8 |  | 1 |
| Cyc/P |  |  |  | 3 |  |  |
| Cyc/Imurek |  |  |  |  |  |  |
| Cyc/Imurek/P |  |  |  | 1 |  | 1 |
| Cyc + x, n (%) | 5 (38.5) | 4 (66.7) | 2 (33.3) | 35 (35.7) | 8 (66.7) | 7 (58.3) |
| Other / unknown, n (%) |  |  |  | 9 (9) | 3 (25%) | 1 (0.08) |

Tac: Tacrolimus, MMF: Mycophenolate Mofetil, Cyc: Cyclosporine, P: Prednisone, FU: Follow up

Suppl. Table 4. Individual characteristics of the pediatric recipients developing *de novo* DSA

|  | Child 1 | Child 2 | Child 3 | Child 4 | Child 5 | Child 6 |
| --- | --- | --- | --- | --- | --- | --- |
| Age at transplantation, years | 3 | 10 | 2 | 3 | 4 | 7 |
| Age at de novo DSA occurrence | 3 | 13 | 6 | 6 | 10 | 10 |
